# Role of fibroblast growth factors in bone regeneration

**DOI:** 10.1186/s41232-017-0043-8

**Published:** 2017-08-01

**Authors:** Pornkawee Charoenlarp, Arun Kumar Rajendran, Sachiko Iseki

**Affiliations:** Section of Molecular Craniofacial Embryology, Tokyo Medical and Dental University Graduate School of Medical and Dental Sciences, 1-5-45 Yushima, Bunkyo-ku, Tokyo, 113-8549 Japan

**Keywords:** Bone regeneration, FGFs, FGF2, FGF9, FGF18, Osteogenesis, Tissue engineering

## Abstract

Bone is a metabolically active organ that undergoes continuous remodeling throughout life. However, many complex skeletal defects such as large traumatic bone defects or extensive bone loss after tumor resection may cause failure of bone healing. Effective therapies for these conditions typically employ combinations of cells, scaffolds, and bioactive factors. In this review, we pay attention to one of the three factors required for regeneration of bone, bioactive factors, especially the fibroblast growth factor (FGF) family. This family is composed of 22 members and associated with various biological functions including skeletal formation. Based on the phenotypes of genetically modified mice and spatio-temporal expression levels during bone fracture healing, FGF2, FGF9, and FGF18 are regarded as possible candidates useful for bone regeneration. The role of these candidate FGFs in bone regeneration is also discussed in this review.

## Background

Tissue engineering is an interdisciplinary field of research and clinical applications, which focuses on restoration of impaired function and morphology of tissues and organs by repair, replacement, or regeneration. It uses a combination of several technological approaches beyond traditional transplantation and replacement therapies. The key components of these approaches are using of cells, scaffolds, and bioactive factors.

Bone is a specialized connective tissue that is being continuously remodeled throughout life. However, many complex clinical conditions such as large traumatic bone defects, osteomyelitis, tumor resection, or skeletal abnormalities can impair normal bone healing. Bone tissue engineering is required for regenerating tissue from these conditions. Studies on the mechanisms of physiological, pathological skeletal development and fracture healing have provided a wealth of information towards potential methods for regulating osteoblast proliferation and differentiation to regenerate bone.

Here, we focus on one of the main components of tissue engineering, bioactive factors, especially fibroblast growth factors (FGFs) and their roles in bone regeneration.

FGF signaling in skeletal formation has been demonstrated by identification of gain-of-function mutations in human FGF receptor (FGFR) genes in craniosynostosis and dwarfism patients and skeletal phenotypes in genetically modified mice for FGFs and FGFRs [1]. FGFRs are transmembrane tyrosine kinase receptors that belong to the immunoglobulin (Ig) superfamily consisting of extracellular, transmembrane, and intracellular tyrosine kinase domains. Binding of FGFs to FGFRs activates intracellular downstream signaling pathways such as RAS-MAP and PI3K-AKT [2]. The FGFR family consists of four members, FGFR1 to FGFR4. Among the four FGFRs, skeletal mutations have been found in FGFRs1–3 expressed in the osteoblast cell lineage. Most of the mutations are point mutations, and distinct mutation sites result in different syndromes [1]. Some of the mutations have been introduced into mice and confirmed to affect skeletal development.

## FGFs and bone regeneration

The mammalian FGF family contains 22 members. Some of them are intracellular FGFs (iFGFs), FGFs 11–14, which are expected to function without binding to FGFRs. FGF19 (FGF15 for mice), FGF21, and FGF23 are hormone-like FGFs which act in an endocrine manner in postnatal life. All other FGFs have high affinity to heparin and act in a paracrine manner by binding to the four receptors with different levels of affinities [3–5]. The roles of various FGFs are compiled in Table 1. Skeletal phenotypes after deletion of FGFs in mice are found in FGFs 2, 8, 9, 10, 18, and 23 [6], which confirm the indispensable function of FGF/FGFR signaling in the process of osteogenesis. It is of note that FGF/FGFR signaling does not directly induce osteoblast differentiation but is known to modulate osteoblast differentiation. However, the exact mechanism of FGF/FGFR signaling in bone healing or regeneration has not been elucidated. Schmid et al. [7] reported expression levels of different FGFs by reverse transcriptase polymerase chain reaction (RT-PCR) during normal healing of tibial fracture in mice. Throughout the healing process, FGFs 2, 5, and 6 were upregulated with different levels. FGF9 was highly expressed at the early stage of healing. FGFs 16 and 18 were transcribed at the late stage. Upregulation of FGFs 1 and 17 was delayed after callus formation. This study also identified concordance between the expression of the particular FGFs and their known receptors during different stages of fracture repair. Among three FGFRs expressed in the osteoblast cell lineage, FGFR3 showed the greatest change in expression levels. This study provided the idea of how FGFs work at the different stages of healing, which could be applied to bone regenerative therapy.

**Table 1:** List of FGFs and their various functions

Subfamily	FGFs	Manner of action	Prime functions	References
FGF1/2	FGF1	Paracrine	Patterning of optical vesicle	[36]
	FGF2	Paracrine	Neuronal, skeletal, vascular tone; heart repair	[37–39]
FGF4/5/6	FGF4	Paracrine	Proliferation of inner cell mass	[40]
	FGF5	Paracrine	Hair growth cycle regulator	[41]
	FGF6	Paracrine	Regulation of muscle regeneration	[42]
FGF3/7/10/22	FGF3	Paracrine	Inner ear formation, regulation of tooth morphogenesis	[43, 44]
	FGF7	Paracrine	Modulation of hair growth, kidney development	[45, 46]
	FGF10	Paracrine	Regulator of development of many organs such as brain, limb, lung, pancreas	[47, 48]
	FGF22	Paracrine	Presynaptic organization in brain development, hair development	[49, 50]
FGF9/16/20	FGF9	Paracrine	Lung development, maintenance of stemness in nephrons, bone repair, mammalian sex determination	[51–53]
	FGF16	Paracrine	Heart development	[54]
	FGF20	Paracrine	Inner ear development, maintenance of stemness in nephrons	[52, 55]
FGF8/17/18	FGF8	Paracrine	Development of brain, limbs, cardiovascular system, craniofacial region	[56–59]
	FGF17	Paracrine	Brain development	[60]
	FGF18	Paracrine	Bone and cartilage development, lung development	[22, 61]
FGF11/12/13/14	FGF11	Intracrine	Signalling functions during tooth development	[62]
	FGF12	Intracrine	Unclear	
	FGF13	Intracrine	Signaling functions during tooth development	[62]
	FGF14	Intracrine	Regulation of neurotransmission of motor functions	[63]
FGF15/19/21/23	FGF15/19	Endocrine	Regulates hepatic glucose metabolism	[64]
	FGF21	Endocrine	Lipid metabolism regulator	[65]
	FGF23	Endocrine	Phosphate and vitamin D metabolism	[66]

The table shows the subfamilies of various FGFs, FGFs under each subfamily, the manner of action of each FGF, and their prime functions

Considering clinical applications, studies involving modification of FGF signaling by ligands are more practical compared to those involving modulating FGFRs. Animal studies revealed that the expression of FGFs 8 and 10 is required for the early stage of limb development, which suggests that they are not directly involved in osteogenesis. Among FGFs which change their expression levels during bone fracture healing, FGF1 protects the osteoblast cell lineage from cell death [8]. FGF5 is associated with the hair follicle cycle. FGF6 is involved in muscle regeneration and those events that occur during the healing process. Therefore, in this review, we chose FGFs 2, 9, and 18 to discuss about their properties and applications for bone regeneration.

## FGF2

FGF2 is the most common FGF ligand that is being used in the regenerative medicine field including bone regeneration. It has been well known that FGF2 is a critical component of maintenance of many kinds of stem cell cultures [9]. Stabilization of FGF2 levels in a culture medium using polyesters of glycolic and lactic acid (PLGA) microspheres as a FGF2 release controller successfully improved the expression of stem cell markers, increased stem cell numbers, and decreased spontaneous differentiation [10].

*FGF2*-deleted mice showed a significant decrease in bone mass and bone formation without gross abnormalities. Bone marrow stromal cells (BMSCs) from the *FGF2* ^−/−^ mice demonstrated decreased osteoblast differentiation, which can be partially rescued by addition of exogenous FGF2 in vitro [11]. Furthermore, *FGF2* ^−/−^ BMSC-derived osteoblasts displayed a marked reduction in inactive phosphorylated glycogen synthase kinase-3 (GSK-3) as well as a significant decrease in Dkk2 mRNA, which plays important roles in osteoblast differentiation. These results suggested that FGF2 is an endogenous, positive regulator of bone mass [12]. In contrast, non-specific overexpression of FGF2 (Tg-*FGF2*) in mice exhibits a dwarf phenotype with impaired bone mineralization and osteopenia [13]. Addition of FGF2 into a culture medium of a mouse osteoblast-like cell line, MC3T3-E1, activated cell proliferation and suppressed mineralization [14]. In this study, treatment of the cells with FGF10 as an experimental control did not show any effects. These observations suggested that FGF2 could work in both directions for osteogenesis promotion and inhibition. It is important to elucidate conditions for positive and negative osteogeneses.

## FGF9

*FGF9* ^−/−^ mice showed disproportionate shortening of the proximal skeletal elements (rhizomelia), which suggests that FGF9 promotes chondrocyte hypertrophy and vascularization of the cartilage anlagen [15]. A missense mutation of *FGF9* in mice resulted in decreased heparin binding, which caused elbow-knee synostosis [16]. A similar mutation was also found in humans [17]. *FGF9* ^+/−^ mice did not seem to have a particular phenotype. However, bone healing of a 1-mm unicortical defect was impaired with decreased levels of neovascularization and osteoclast recruitment. This condition was rescued by exogenous addition of FGF9 (2 μg) with collagen sponge but not by exogenous FGF2 application [18]. These reports elucidated the specific functions of FGF9 in bone healing.

Bone healing of a 1-mm unicortical defect in diabetic model mice (*db*/*db*) was significantly delayed with decreased levels of osteogenesis marker expressions. Treatment of FGF9 with collagen sponge to the defect in the *db*/*db* mice induced better bone healing [19]. Treatment with FGF9-soaked collagen sponge to mouse circular calvarial bone defects of a diameter of 2 mm showed sufficient bone regeneration in postnatal day 7 (P7) mice but not in postnatal day 60 (P60) mice [20]. Addition of FGF9 with various concentrations into dexamethasone-containing media for inducing osteogenesis of BMSCs and dental pulp stem cells resulted in stimulation of proliferation but not differentiation [21].

## FGF18

Deletion of FGF18 in mice resulted in delayed suture formation, reduced osteoblast lineage cell proliferation, delayed osteoblast differentiation, and perinatal death. The long bones of *FGF18* ^−/−^ mice showed reduced osteoblast differentiation but increased chondrocyte proliferation and differentiation. These results suggested that FGF18 demonstrated a positive effect on osteogenesis by enhancing cell proliferation and differentiation but a negative effect on chondrogenesis [22, 23]. However, it was also proposed that FGF18 transduced the signal through FGFR3 to enhance cartilage formation [24].

In vitro analysis on mesenchymal stem cells (MSCs) derived from the bone marrow suggested that FGF18 enhanced osteoblast differentiation by activation of FGFR1 or FGFR2 signaling [25]. They also showed that overexpression of FGF18 by lentiviral infection or direct addition of FGF18 into the culture medium could induce the expression of osteoblast marker genes in C3H10T1/2 fibroblastic cells. Treatment of FGF18 on rat-derived MSCs under a differentiation-inducing condition showed elevated expression of osteoblast differentiation markers and mineralization [26]. Low-dose FGF18 treatment with bone morphogenetic protein 2 (BMP2)-dependent osteogenic induction of MC3T3-E1 cells enhanced mineralization whereas high-dose treatment inhibited the process (unpublished observation of Sachiko Iseki). FGF18-soaked heparin-coated acrylic beads accelerated osteoblast differentiation in mouse fetuses by upregulating the expression of BMP2 in osteoblast cell lineage cells [27]. In accordance with the above reports, FGF18 application with BMP2 in cholesteryl group- and acryloyl group-bearing pullulan (CHPOA) nanogels stabilized BMP2-dependent bone regeneration of critical-sized bone defects on mouse calvarium [28].

## Application of FGFs in bone regeneration

The above discussions suggest that although FGFs do not have osteoinductive property, they function as an accelerator of osteogenesis under the appropriate conditions. It is possible that FGF2 and FGF9 work on proliferation of osteoblast cell lineage as well as induction of angiogenesis, and FGF18 functions in promotion of osteoblast differentiation. Tables 2 and 3 show some of the in vivo experiments in which FGFs were applied to non-critical- and critical-sized bone defects for bone healing, respectively. Further applications of FGFs have been elaborated by Du et al. and Gothard et al. [29, 30].

**Table 2:** Application of different FGFs in non-critical-sized bone defect in vivo models

Growth factor	Dose	In vivo model	Carrier	Investigations	Effect	References
FGF2	200 μg	Monkey ulna fracture	Injectable gelatin hydrogel	Bone mineral content and mechanical properties	Accelerates fracture healing and prevents nonunion	[67]
FGF2	2.5 μg	Rat periodontal defect (2 × 2 × 1.7 mm)	Injectable calcium phosphate cement	Histology and histomorphometry of bone	Increased periodontal regeneration	[68]
FGF2	50 μg	Rat calvarial defect (4-mm diameter)	PLGA/β-TCP	Histomorphometry of bone	Enhanced bone regeneration	[69]
FGF2	50 μg/ml	Rat calvarial defect (5-mm diameter)	Collagen and nano-bioactive glass hybrid membrane	Histomorphometry of bone	Accelerated bone regeneration	[70]
FGF2	45 μg	Rabbit femoral condyle (4-mm diameter and 6 mm long)	Hydrogel polymer	Bone mass and microarchitecture	Enhanced bone regeneration	[32]
FGF2	10 μg	Rat tibia (2-mm diameter, 4 mm long)	Titanium implant	Bone histomorphometry	Synergistically enhanced new bone formation	[71]
Melatonin	100 mg/kg i.p.					
FGF2	200, 400, or 800 μg	Human tibia (high tibial osteotomy)	Gelatin hydrogel	Radiographic assessment of bone	Dose dependently accelerated bone union	[72]
FGF2	100 μg	Rabbit femoral condyle (10 mm^2^ × 5 mm depth)	Interconnected porous calcium hydroxyapatite ceramic	Bone histomorphometry	Decreases lamellar bone formation, increases vascularization and osseointegration	[73]
FGF2	0, 25, or 250 ng	Rat calvarial defect (3.5-mm diameter)	PLGA/gelatin	Radiological, histological, and biochemical examination	Low-dose administration enhanced the degree of calcification and ALP activity	[74]
BMP2	0.1 mg/ml					
FGF9	2 μg	Mouse tibia (1-mm defect)	Collagen sponge	Bone histomorphometry	Enhances angiogenesis and bone regeneration	[19]

The table shows the various growth factors and their combinations used for regeneration of non-critical-sized defects, their dose, the site of application, the carrier used for the application, and the investigations through which the effects of bone healing have been studied

*i.p.* intraperitoneal injection

**Table 3:** Application of different FGFs in critical-sized bone defect in vivo models

Growth factor	Dose	In vivo model	Carrier	Investigations	Effect	References
FGF2	5 ng	Mouse calvarial defect (3.5-mm diameter)	Col-HA/PEG hydrogel	Micro CT and histology of bone	Enhanced bone regeneration	[34]
BMP2	2 μg					
FGF2	10 ng, 100 μg, and 1 μg	Rat mandibular defect (5-mm diameter)	Collagen sponge	Radiological and histological examination	Promotes osteogenesis	[75]
FGF2	200 μg	Beagle dog periodontal defect (6 × 5 mm: vertical × horizontal)	β-TCP	Bone histomorphometry	Enhances formation of new bone and cementum	[76]
FGF18	0.5 μg	Mouse calvaria (3-mm diameter)	CHPOA/hydrogel	Micro CT assessment of bone	Synergistically enhanced new bone formation	[28]
BMP2	0.5 μg					
FGF2 or FGF9 or FGF18	250 ng (P7 mice) or 2.5 μg (P60 mice)	Mouse calvaria defect (2-mm diameter)	Collagen sponge	Micro CT assessment of bone	All FGF ligands promote healing rate in P7 mice. Only FGF18 promotes healing rate in P60 mice	[20]

The table shows the various growth factors and their combinations used for regeneration of critical-sized defects, their dose, the site of application, the carrier used for the application, and the investigations through which the effects of bone healing have been studied

Systemic or subcutaneous injections of FGF2 could enhance osteogenesis. However, it was shown that systemic injections of FGF2 caused adverse extraskeletal effects [31]. Therefore, local administration has been chosen as a more preferable method for applying bioactive factors. FGF2 has been used for inducing angiogenesis and enhancing osteogenesis in non-critical-sized bone defects by activating proliferation of osteoblast cell lineages. FGF9 is also suggested to be involved in angiogenesis by controlling VEGFa expression [18]. As long as osteogenesis is taking place to recover the bone defect, FGF2 can support or even enhance the healing. Recent studies suggest that high-dose FGF2 inhibits progression of osteoblast differentiation [20, 32, 33] (also unpublished observation of Sachiko Iseki) and low concentration of FGF2 enhanced osteogenesis [33, 34]. In contrast, it is likely that high-dose FGF18 can promote osteoblast differentiation in vivo [20, 28], while FGF18 treatment in vitro inhibits mineralization [14].

Kang et al. developed a sequential delivery system with fiber scaffolds in which FGF2 was released first and then FGF18 [35]. Applying this scaffold to rat calvarial critical-sized bone defects resulted in better bone volume and density, although the amount of FGFs applied to the defect was not clear. This study suggested that it is critical to control the amount or release speed of soluble factors for the bone regeneration process.

## Conclusions

FGFs play an important role in the development and regeneration of various tissues. In this article, we have summarized the prime functions of all FGFs, and further, we have discussed elaborately about FGFs 2, 9 and 18, which play a major role in bone regeneration. We have also discussed about different carrier systems for FGF delivery in different animal models for bone regeneration. With the ongoing advancements in the field of cellular and molecular biology, we could expect that more detailed functioning of FGF/FGFR will be elucidated. Further, with the advent of novel carriers and protein delivery systems, it could be possible that the spatio-temporal release of FGFs can be controlled precisely as needed. This would improve our understanding and help us to clinically translate the use of FGFs to achieve effective bone regeneration.

## Abbreviations

BMP2: Bone morphogenetic protein 2
BMSC: Bone marrow stromal cells
CHPOA: Cholesteryl group- and acryloyl group-bearing pullulan
Col-HA/PEG: Collagen-hydroxyapatite/polyethylene glycol
Dkk2: Dickkopf-related protein 2
FGF: Fibroblast growth factor
FGFR: Fibroblast growth factor receptor
GSK-3: Glycogen synthase kinase-3
iFGF: Intracellular fibroblast growth factor
Ig: Immunoglobulin
MSC: Mesenchymal stem cells
PLGA: Polyesters of glycolic and lactic acid
RT-PCR: Reverse transcriptase polymerase chain reaction
Tg-FGF2: Transgenic fibroblast growth factor 2
VEGFa: Vascular endothelial growth factor A
β-TCP: Beta tricalcium phosphate
